# Environmental Sustainability-Related Knowledge, Behaviors, and Perceived Barriers Among Italian Intensive Care Unit Nurses: A Cross-Sectional Survey

**DOI:** 10.3390/healthcare14183031

**Published:** 2026-09-16

**Authors:** Francesca Trotta, Luciano Midolo, Davide Bartoli, Ercole Vellone, Rosaria Alvaro, Marco Di Muzio, Gianluca Pucciarelli, Francesco Petrosino

**Affiliations:** 1 Department of Health Professions, Sant’Andrea University Hospital, 00189 Rome, Italy; francesca.trotta@uniroma1.it; 2 Department of Biomedicine and Prevention, University of Rome Tor Vergata, 00133 Rome, Italy; luciano.midolo@students.uniroma2.eu (L.M.); ercole.vellone@uniroma2.it (E.V.); rosaria.alvaro@uniroma2.eu (R.A.); gianluca.pucciarelli@uniroma2.eu (G.P.); 3 Department of Well-Being, Health and Environmental Sustainability (BeSSA Department), Sapienza University, 00185 Rome, Italy; marco.dimuzio@uniroma1.it; 4 Division of Research Methodology, Department of Nursing, Faculty of Nursing and Midwifery, Wroclaw Medical University, 51-618 Wroclaw, Poland; 5 Health Professions Directorate, Local Health Authority Rome 3, 00125 Rome, Italy; f.petrosino.75@gmail.com

**Keywords:** environmental sustainability, intensive care units, critical care nursing, climate change, sustainable healthcare

## Abstract

**Highlights:**

**What are the main findings?**

- Italian Intensive Care Unit (ICU) nurses reported high climate change knowledge, but only moderate engagement in sustainability-related behaviors within intensive care settings.
- Workload, time pressure, and limited organizational support were frequently reported as perceived barriers to sustainable practice in intensive care settings.

**What are the implications of the main findings?**

- The findings identify both educational and organizational conditions as areas warranting evaluation in future sustainability interventions.
- The limited reported awareness of several organizational initiatives highlights the need for further investigation of their implementation and communication within ICU settings.

**Abstract:**

**Background/Objectives**: Healthcare systems are increasingly recognized as contributors to climate change, and intensive care units represent particularly resource-intensive clinical environments. Nurses play a central role in shaping daily care practices that may influence the environmental footprint of critical care. However, limited quantitative evidence is available on intensive care nurses’ knowledge, behaviors, and perceived barriers regarding environmental sustainability. This study aimed to assess knowledge, sustainability-related behaviors, and perceived barriers among Italian intensive care unit nurses using the preliminary version of the Knowledge, Attitudes, and Behaviors Questionnaire on Environmental Sustainability in Intensive Care Units (KABQES-ICU). Despite the instrument’s name, attitudes were not operationalized as a separate scored domain in the preliminary version used in this study and were therefore not analyzed as an independent construct. **Methods**: A cross-sectional online survey using self-selected convenience sampling was conducted between November 2024 and July 2025. Participants were Italian registered nurses or nurse coordinators working in intensive care settings with at least one year of critical care experience. The survey was distributed through the official website of the Italian National Association of Critical Care Nurses. Descriptive statistics, correlation analyses, and group comparisons were performed on a final analytical sample of 278 participants. **Results**: Participants reported high levels of climate change knowledge, while sustainability-related behaviors were moderate, particularly in workplace settings. Perceived workload, time pressure, and organizational barriers were moderate to high, reflecting substantial perceived obstacles to the implementation of sustainable practices in everyday intensive care. Relatively small proportions of participants reported awareness of specific organizational sustainability initiatives, particularly carbon-emissions reporting, structured emission-reduction plans, staff training, and sustainable procurement strategies. Knowledge was positively but weakly associated with home- and work-based sustainability behaviors, with these associations showing small effect sizes. Sustainability-related scores were only weakly associated with sociodemographic and professional characteristics. **Conclusions**: Italian intensive care nurses reported high climate change knowledge scores alongside only moderate levels of workplace sustainability behaviors. The modest unadjusted associations indicate that climate change knowledge was only weakly related to reported workplace sustainability behaviors. These exploratory findings should not be interpreted as evidence of independent predictors or of the relative contribution of individual and organizational factors.

## 1. Introduction

Healthcare systems are increasingly recognized as important contributors to global climate change, accounting for approximately 4–5% of worldwide greenhouse gas emissions [1]. Within this context, Intensive Care Units (ICUs) represent one of the most resource-intensive environments in modern hospitals. Their continuous energy consumption, reliance on technologically advanced life-support systems, and extensive use of single-use materials generate substantial environmental burdens across the entire lifecycle of care [2]. Energy demands associated with heating, ventilation and air-conditioning systems, continuous monitoring equipment, and highly specialized devices, together with the production, transport, and disposal of pharmaceuticals, personal protective equipment, and disposable medical devices, contribute significantly to the ecological footprint of intensive care [3]. Estimates suggest that a substantial proportion of healthcare-related emissions originates from supply chains linked to these materials, highlighting the systemic nature of environmental impact in critical care [4].

The urgency of addressing environmental sustainability in healthcare is therefore no longer limited to environmental policy discussions but has become an ethical and professional imperative for clinical practice. The environmental consequences of healthcare delivery are increasingly recognized as intertwined with patient safety, resource stewardship, and long-term health system resilience [5]. From this perspective, the sustainability of intensive care does not merely concern technological innovation or infrastructure redesign; rather, it also depends on how healthcare professionals interpret environmental responsibility and translate it into everyday clinical behaviors [6].

Among healthcare professionals, nurses occupy a particularly strategic position in this transformation. As the largest professional group in healthcare and the frontline professionals most consistently present at the bedside, nurses influence a wide range of decisions that collectively shape the environmental impact of ICU practice [6,7]. Daily actions such as the selection and management of materials, avoidance of unnecessary disposables, optimization of equipment use, and environmental resource stewardship accumulate into measurable effects on the ecological footprint of critical care units. These behaviors, however, may be related to professional knowledge, ethical values, and organizational contexts that are relevant to how sustainability is interpreted and prioritized within clinical routines [8].

Environmentally sustainable healthcare practices may be associated with professionals’ knowledge, behaviors, and the contextual conditions in which they operate, which together shape how sustainability principles are understood and translated into everyday clinical practice [9,10]. Knowledge may support the recognition of sustainable alternatives [11], whereas reported behaviors reflect their implementation in practice [12]. Perceived workload, time pressure, organizational limitations, and lack of support may also be associated with the incorporation of sustainable behaviors into routine clinical care [13]. Together, these domains form a multidimensional framework through which sustainable healthcare practices can be understood and strengthened.

Despite growing recognition of sustainability as a healthcare priority, empirical evidence examining these factors in intensive care remains limited. Existing research has primarily focused on technological or organizational strategies, with comparatively less attention to frontline clinicians’ perspectives and practices [14]. This gap is particularly relevant in ICUs, where clinical complexity, urgent decision-making, and infection-control demands may uniquely influence sustainability-related behaviors.

Previous studies examining healthcare professionals’ perspectives have also presented important methodological limitations. Qualitative studies have provided detailed insights into nurses’ experiences, professional responsibilities, and perceived barriers, but they cannot quantify the distribution of these characteristics within larger populations [7,8]. Conversely, available quantitative surveys have frequently included heterogeneous groups of healthcare professionals or have not specifically addressed the clinical and organizational characteristics of intensive care [14,15]. Consequently, evidence remains limited regarding the extent to which ICU nurses’ knowledge is associated with their sustainability-related behaviors and how reported workload and organizational barriers are related to these practices.

Furthermore, at the time of data collection, the absence of context-specific measurement tools represented a critical methodological limitation in the literature. Most available instruments assessing environmental sustainability in healthcare have been designed for general healthcare populations and do not adequately capture the unique organizational, technological, and ethical dynamics of intensive care environments [15,16]. As a result, the extent to which ICU professionals possess the knowledge and behavioral readiness required to integrate sustainability into routine practice remains largely unknown.

Systematically assessing these dimensions is a necessary step for advancing sustainable critical care. Understanding the current level of knowledge among ICU nurses, the sustainability-related behaviors implemented in personal and professional settings, and the barriers that may hinder their implementation can provide essential insights for the development of targeted educational programs, organizational policies, and behavior-change interventions [16]. Without such empirical evidence, sustainability strategies risk remaining predominantly top-down initiatives that fail to resonate with the realities of frontline clinical work [17,18].

From a policy and organizational perspective, this assessment may help determine whether sustainability interventions should primarily focus on professional education or whether they should also address staffing, workload, clinical workflows, infrastructure, procurement, leadership support, and the communication of existing sustainability initiatives. Although a cross-sectional survey cannot establish the effectiveness of specific policies, it can provide baseline evidence for identifying priorities and designing future organizational interventions.

In response to this gap, the present study used a preliminary version of the Knowledge, Attitudes, and Behaviors Questionnaire on Environmental Sustainability in Intensive Care Units (KABQES-ICU), which was under development at the time of data collection [16]. The questionnaire was designed to assess climate change knowledge, home- and work-based sustainability behaviors, and perceived barriers to sustainable clinical practice among ICU nurses. Following the initial submission of the present study, the KABQES-ICU underwent formal psychometric validation, which supported a five-factor structure comprising climate change knowledge, home-based sustainability behaviors, work-based sustainability behaviors, workload and time-pressure barriers, and organizational and support barriers [19]. However, the data reported in the present study were collected using the preliminary version of the instrument, and this distinction is maintained throughout the manuscript.

The novelty of the present study lies in providing an exploratory baseline assessment of sustainability-related knowledge, behaviors, perceived barriers, and reported organizational initiatives among Italian ICU nurses.

The aim of this study was to assess climate change knowledge, home- and work-based sustainability behaviors, and perceived barriers related to environmental sustainability among Italian ICU nurses using the preliminary version of the KABQES-ICU instrument.

The study addressed the following research questions:

1. What levels of climate change knowledge, home- and work-based sustainability behaviors, and perceived barriers are reported by Italian ICU nurses?
2. What associations exist among climate change knowledge, home-based sustainability behaviors, work-based sustainability behaviors, and perceived barriers?
3. Do sustainability-related knowledge, behaviors, and perceived barriers differ according to nurses’ sociodemographic and professional characteristics?
4. What organizational sustainability initiatives are participants aware of as being present within their workplace organizations?

These research questions were informed by the M-COM-B framework [20] and by previous evidence concerning sustainability-related knowledge, education, and the possible consistency of pro-environmental behaviors across home and workplace settings [10,11,21,22]. However, the study was designed as a descriptive and exploratory baseline assessment rather than as a confirmatory test of a prespecified theoretical model. Accordingly, the correlation and subgroup analyses were interpreted as exploratory and hypothesis-generating.

## 2. Materials and Methods

### 2.1. Design

A cross-sectional survey design was employed to provide a comprehensive snapshot of how environmental sustainability is currently understood and enacted among Italian ICU nurses. This study was designed and reported in accordance with the STROBE (Strengthening the Reporting of Observational Studies in Epidemiology) guidelines [23]. By generating empirical evidence on these dimensions, the study sought to contribute to the development of evidence-informed strategies capable of embedding environmental sustainability into the culture and daily practice of intensive care.

The study was designed as an exploratory baseline assessment of sustainability-related knowledge, behaviors, perceived barriers, and organizational initiatives within the Italian ICU nursing context. It was not designed to develop a predictive model or to establish causal relationships between the variables examined.

### 2.2. Sampling

The target population comprised registered nurses and nurse coordinators working in intensive care settings throughout Italy. Participants were eligible if they: (1) were registered bedside nurses or nurse coordinators working in an intensive care setting; and (2) had at least one year of professional experience in critical care.

A self-selected convenience sampling approach was adopted. Eligibility criteria defined the population of interest; however, participants were not deliberately selected by the researchers and independently chose whether to participate through the open-access survey link. The sampling strategy was therefore classified as self-selected convenience sampling rather than purposive sampling.

A total of 309 nurses participated across the pilot and main survey phases. The first 31 participants were involved in pilot testing and were excluded from the main analysis, resulting in a final analytical sample of 278 participants. No formal a priori sample-size or precision calculation was undertaken before recruitment. The final sample size was therefore not based on a predetermined statistical target. Recruitment was continued throughout the predefined data-collection period, and all eligible responses obtained during the main survey administration were included, resulting in an achieved analytical sample of 278 participants. Consequently, the study was not designed to guarantee a prespecified level of precision or statistical power for the secondary correlation and subgroup analyses.

Because the survey was disseminated through an open-access link and no exhaustive sampling frame was available, the exact size of the accessible population and the number of eligible ICU nurses who received or viewed the invitation could not be determined. Consequently, a conventional response rate could not be calculated, and the formal representativeness of the sample could not be assessed.

### 2.3. Instrument

The survey was conducted using the preliminary version of the Knowledge, Attitudes, and Behaviors Questionnaire on Environmental Sustainability in Intensive Care Units (KABQES-ICU), a self-reported, multidimensional instrument developed to assess sustainability-related knowledge, behaviors, and perceived barriers among ICU nurses [19].

Item generation was informed by previous qualitative research on environmental sustainability in intensive care [8] and by the Modified Capability, Opportunity, Motivation–Behavior conceptual model [20]. The items were subsequently refined through expert review.

The items of the preliminary KABQES-ICU were generated specifically for this research programme and were not directly reproduced from an existing questionnaire. Item development was informed by findings from a previous qualitative study of critical care nurses’ experiences of environmental sustainability [8], the Modified Capability, Opportunity, Motivation–Behavior framework [20], and the relevant literature on environmental sustainability in intensive care and nursing practice [13,15,24,25]. Climate change knowledge items were developed to assess familiarity with the principal relationships between healthcare, climate change, and environmental sustainability. Home- and work-based behavior items reflected sustainability-related actions identified in the literature and in the previous qualitative study, whereas the perceived-barrier items operationalized workload, time pressure, resource availability, organizational infrastructure, leadership, and professional support. All items were subsequently evaluated and refined through expert review and pilot testing.

The complete wording, response options, scoring procedure, and conceptual or empirical provenance of the 33 items included in the preliminary version administered in the present study are reported in Appendix A (Table A1). These items should be distinguished from the 28-item, five-factor version subsequently retained in the formal psychometric validation study [19].

The questionnaire comprised two sections: (1) a sociodemographic section and (2) a psychometric section provisionally organized into four domains.

Sociodemographic variables include age, gender, geographic area, professional role, educational level, and years of professional and ICU experience. Educational qualifications were assessed as non-mutually exclusive categories because participants could report more than one completed qualification.

The preliminary psychometric section comprises 33 items distributed across four domains: climate change knowledge (5 items), home-based sustainability behaviors (5 items), work-based sustainability behaviors (8 items), and workload and time-pressure, organizational and support barriers (15 items). Items are rated on 5-point Likert-type scales, with response options varying according to the construct measured: knowledge (0 = “not at all familiar” to 4 = “extremely familiar”), behaviors (0 = “never” to 4 = “always”), and perceived barriers (0 = “not at all” to 4 = “extremely”). Higher scores indicate greater levels of the respective construct, and domain scores were calculated by summing item responses within each subscale.

The questionnaire also included a descriptive multiple-response checklist of 19 organizational sustainability initiatives. Participants were asked to select all initiatives they were aware of as being present in their workplace organization. The checklist also included the option “I am not aware of any sustainability-related initiatives”. Non-selection of an initiative was not interpreted as evidence that the initiative was absent. These items were used solely to characterize participants’ reported awareness of the organizational context and did not constitute a validated organizational scale or composite exposure measure.

Before the main survey administration, the preliminary questionnaire was pilot-tested with 31 nurses working in an adult ICU to assess item clarity, comprehensibility, and survey functionality. Participants’ feedback was used to refine the wording and presentation of the questionnaire. The pilot sample was excluded from the main analysis.

At the time of data collection and the original analysis, the KABQES-ICU had not yet undergone formal psychometric validation. Following the original submission of the present manuscript, a separate psychometric evaluation was published using the same main survey sample but addressing a distinct methodological objective. The psychometric evaluation supported a refined 28-item, five-factor structure comprising climate change knowledge, home-based sustainability behaviors, work-based sustainability behaviors, workload and time-pressure barriers, and organizational and support barriers. The five-factor solution explained 58.9% of the total variance. Confirmatory factor analysis showed acceptable-to-good model fit (CFI = 0.932–0.944; TLI = 0.925–0.937; RMSEA = 0.059–0.064), while internal consistency was satisfactory across domains (Cronbach’s α = 0.74–0.86; composite reliability > 0.78). All standardized CFA factor loadings were statistically significant and ranged from 0.47 to 0.91. Five items were removed during the iterative psychometric refinement, resulting in the final 28-item structure [19].

The present study retains the preliminary 33-item, four-domain scoring structure used for the original exploratory baseline analyses. Accordingly, the findings should be interpreted as descriptive evidence generated using the preliminary version of the instrument rather than the subsequently refined version.

Although the name KABQES-ICU includes the term “Attitudes”, the preliminary version did not operationalize attitudes as a separate scored domain. Attitudes were therefore not analyzed as an independent construct in the present study.

### 2.4. Data Collection

Data were collected through a web-based survey administered between November 2024 and July 2025. The open-access survey link was disseminated through the official website of the Italian National Association of Critical Care Nurses (ANIARTI) and promoted through ANIARTI’s social media channels and the research group’s social media channels. No formal reminders were issued during the recruitment period. Access was not based on personalized invitations and was not technically restricted to individually verified eligible participants; eligibility was assessed through participants’ self-reported professional characteristics. The survey could be completed anonymously through a secure web-based platform. The average completion time ranged from approximately 10 to 25 min.

Upon completion of data collection, responses were exported into a spreadsheet format and subsequently prepared for statistical analysis.

The survey platform did not record aggregate information on the number of individuals potentially reached, those who viewed the invitation, accessed the survey, provided consent, started the questionnaire, or discontinued it before submission. Therefore, these stages of participant flow could not be quantified, and a conventional response rate could not be calculated.

To reduce the potential for common method bias, participation was anonymous, and different response anchors were used across the knowledge, behavior, and perceived-barrier domains. Nevertheless, because all constructs were assessed using the same self-report survey, residual common method bias could not be excluded.

### 2.5. Ethical Considerations

The study was conducted in accordance with the principles of the Declaration of Helsinki. Ethical approval was obtained from the local Ethics Committee “Campania Sud” (protocol n. 124_r.p.s.o.).

Participation was voluntary and anonymous. Prior to accessing the questionnaire, participants were provided with detailed information about the study aims, procedures, and data handling. Electronic informed consent was obtained from all participants before data collection.

No personally identifiable information was collected, and all data were handled confidentially and stored in accordance with applicable data protection regulations.

### 2.6. Statistical Analysis

All analyses were conducted on the full sample of 278 intensive care nurses included in the main survey. No missing data were observed for the 33 questionnaire items used to calculate the four domain scores; therefore, all four domain scores were available for all 278 participants. Missing data were limited to selected sociodemographic variables and were handled using available-case analysis. No imputation was performed. Descriptive statistics were first computed for all sociodemographic and professional variables (frequencies and percentages for categorical variables; mean, standard deviation, minimum and maximum for continuous variables) and for each of the four composite domain scores, which were rescaled to a 0–100 metric. Distributional properties (skewness and kurtosis with standard errors) were examined to assess approximate normality and to justify the use of parametric procedures.

Composite domain scores were then derived for Climate Change Knowledge, Home-Based Sustainability Behaviors, Work-Based Sustainability Behaviors, and Workload, Time Pressure, Organizational, and Support Barriers.

Organizational sustainability initiatives were analyzed descriptively using the number and percentage of participants selecting each response option. Multiple responses were allowed. Because non-selection could reflect either absence of an initiative or lack of participant awareness, complementary counts were not interpreted as indicating that an initiative was absent. The checklist items were not combined into a composite score or included in inferential analyses.

Bivariate associations between domain scores were examined using Pearson and Spearman correlation coefficients, with two-tailed tests and a significance threshold of *p* < 0.05 [26]. To explore relationships between the sustainability dimensions and sociodemographic or professional characteristics, we used one-way analysis of variance (ANOVA) and tests for linear trends. For these analyses, the four composite domain scores were treated as dependent variables, while gender identity, age, professional role, educational qualification, years of nursing experience, and years of ICU experience were treated as grouping or explanatory variables, as appropriate. Geographic distribution was summarized descriptively and was not included in inferential group comparisons because several individual regions were represented by very small numbers of participants. Where omnibus F tests were statistically significant, the pattern of group differences and linear components was interpreted in light of effect sizes and the underlying theoretical framework [26]. The study was primarily descriptive and exploratory. Correlation and group-comparison analyses were conducted as secondary, unadjusted analyses intended to characterize patterns within the participating sample rather than to estimate independent associations or identify predictors. Given the exploratory nature of the secondary analyses, no formal adjustment for multiple comparisons was applied. Accordingly, *p*-values were interpreted cautiously alongside effect sizes, and statistically significant subgroup findings were considered hypothesis-generating. No multivariable regression or predictive models were specified or fitted, and the analyses were not designed to establish causal relationships. All statistical analyses were performed using IBM SPSS Statistics, version 26.0 (IBM Corp., Armonk, NY, USA). All tests were two-sided, and statistical significance was set at *p* < 0.05.

## 3. Results

### 3.1. Sample Characteristics

A total of 278 intensive care nurses completed the survey and were included in the analyses. All four domain scores were available for all 278 participants. Missing data were limited to selected sociodemographic variables: gender identity was missing for 4 participants (1.4%), professional role for 3 participants (1.1%), and geographic region for 2 participants (0.7%), as reported in Table 1. Consequently, the effective sample size varied only for analyses involving these variables. Gender identity was reported as male by 93 participants (33.5%), female by 180 (64.7%), and non-binary by 1 (0.4%); 4 participants (1.4%) did not declare their gender identity. Participants’ age ranged from 24 to 65 years (M = 40.47, SD = 10.32), reflecting an overall middle-aged and professionally mature workforce.

All respondents reported living in Italy, with a broad distribution across regions. The most represented regions were Lazio (20.5%), Toscana (12.2%), Lombardia (11.2%), Piemonte (10.8%), Emilia-Romagna (10.4%) and Veneto (8.6%), while the remaining regions contributed smaller proportions, with participants from several areas of the country. Regarding educational qualifications, 53% of participants reported holding a three-year bachelor’s degree, 16% a master’s degree, 43% a first-level postgraduate master, 4% a second-level postgraduate master, and 2% a PhD. Because participants could report more than one completed qualification, these categories were not mutually exclusive. Regarding professional role, 243 (87.4%) were clinical nurses and 32 (11.5%) were nurse coordinators, and 3 (1.1%) did not declare their role, highlighting the predominance of the clinical nurse profile within the sample. Overall clinical experience as a registered nurse ranged from 1 to 40 years (M = 16.05, SD = 10.15), while experience specifically in intensive care ranged from 1 to 38 years (M = 10.98, SD = 9.23), indicating substantial exposure to the intensive care setting for a large proportion of participants. The four domain scores were available for all 278 participants.

### 3.2. Participant-Reported Organizational Sustainability Initiatives

Participant-reported awareness of organizational sustainability initiatives varied considerably across checklist items (Table 2). The most frequently selected initiatives were sterilization and reuse of metal instruments and other devices (n = 134, 48.2%) and digitization of health records (n = 129, 46.4%). In contrast, annual carbon-emissions reporting and selection of suppliers engaged in decarbonization were each selected by 5 participants (1.8%), while 85 participants (30.6%) reported that they were not aware of any sustainability-related initiatives. These findings represent participants’ reported awareness only and should not be interpreted as objective estimates of initiative implementation or absence within healthcare organizations.

### 3.3. Domain Score Distributions

Descriptive statistics for the four domain scores are presented in Table 3. Because the domain scores were available for all 278 participants, no participant was excluded from the descriptive analyses of the domain scores. At the composite level, all domain scores were standardized on a 0–100 scale and showed good variability across respondents. The Climate Change Knowledge score (CCK) ranged from 20.00 to 100.00 (M = 79.68, SD = 16.21), indicating generally high levels of climate-related knowledge in the sample. Home-Based Sustainability Behaviors (SB_Home) ranged from 18.75 to 100.00 (M = 63.20, SD = 15.56), suggesting moderately high but heterogeneous engagement in personal sustainable practices. Work-Based Sustainability Behaviors (SB_Work) ranged from 0.00 to 100.00 (M = 59.63, SD = 15.59), reflecting intermediate levels of sustainability-related behaviors perceived and enacted within ICU settings. Workload, Time Pressure, Organizational & Support Barriers (WTPOSB) ranged from 0.00 to 100.00 (M = 63.18, SD = 18.65), pointing overall to moderate to high perceived obstacles at both workload and organizational levels, with substantial interindividual variability.

The distributions of domain scores were approximately normal for all dimensions, with only mild deviations from symmetry and tail weight. CCK showed a moderate negative skew (skewness = −1.06, SE = 0.15) and positive kurtosis (kurtosis = 1.43, SE = 0.29), indicating a concentration of higher knowledge scores and a slightly more peaked distribution. The remaining scales exhibited small absolute skewness values and kurtosis close to zero, suggesting distributions compatible with the assumptions of parametric analyses.

### 3.4. Relationships Between Domain Scores

As shown in Table 4, Pearson correlations showed that higher CCK was weakly associated with more frequent sustainability behaviors at home (r = 0.22, *p* < 0.001) and at work (r = 0.23, *p* < 0.001), as well as with higher perceived workload, time pressure, organizational, and support barriers (r = 0.15, *p* = 0.01). Home-based and work-based sustainability behaviors were moderately correlated (r = 0.55, *p* < 0.001), representing the strongest association among the study variables. Perceived workload, time pressure, organizational, and support barriers were weakly associated with both home-based (r = 0.14, *p* = 0.02) and work-based sustainability behaviors (r = 0.14, *p* = 0.02). Spearman’s rho coefficients showed a similar pattern and comparable effect sizes.

Associations between the four domain scores and sociodemographic or professional characteristics were generally small in magnitude, indicating that sustainability-related cognitions and behaviors were only weakly patterned by background variables. CCK showed weak positive correlations with age (r = 0.14, *p* = 0.02) and years of nursing experience (r = 0.15, *p* = 0.02), while no significant association emerged with gender identity or professional role. Home-based and work-based sustainability behaviors were modestly related to age (r = 0.24, *p* < 0.001; r = 0.22, *p* < 0.001) and professional experience, indicating that older and more experienced nurses tended to report somewhat greater engagement in sustainable practices both at home and in the ICU. Perceived workload, time pressure, organizational, and support barriers showed only small associations with individual and professional characteristics. Overall, these results suggest that, beyond modest age- and experience-related trends, environmental sustainability knowledge, behaviors, and perceived barriers are broadly shared across demographic and educational subgroups of ICU nurses.

### 3.5. Associations with Sociodemographic and Professional Characteristics

Group comparisons were conducted using one-way ANOVA for categorical variables. Effect sizes were estimated using partial eta squared, and the effective sample size was determined separately for each analysis. Group comparisons showed that sustainability-related scores were largely homogeneous across most sociodemographic subgroups, with only a few statistically significant differences in small magnitude. No statistically significant differences in any domain score were observed in the analyses of gender identity (all *p* values ≥ 0.16). The non-binary participant was not included in inferential group comparisons because this category contained only one participant.

Professional role was not significantly associated with Climate Change Knowledge, Home-Based Sustainability Behaviors, or Work-Based Sustainability Behaviors. However, nurse coordinators reported lower perceived workload, time-pressure, organizational, and support barriers than clinical nurses, F(1, 273) = 5.57, *p* = 0.019, ηp^2^ = 0.020. The effect size was small, indicating that professional role accounted for only a limited proportion of variability in perceived barriers.

Educational attainment showed limited associations with the domain scores. Possession of a three-year bachelor’s degree was associated with Work-Based Sustainability Behaviors, F(1, 276) = 4.82, *p* = 0.029, ηp^2^ = 0.017. No statistically significant associations were observed for first-level postgraduate master’s training, second-level postgraduate master’s training or doctoral education. The observed effect sizes were small, indicating that educational variables accounted for only a limited proportion of the variability in the domain scores.

Although some comparisons reached conventional statistical significance, their effect sizes were small (e.g., partial ηp^2^ values around 0.02), indicating limited substantive differences between groups. Accordingly, statistical significance should not be interpreted as evidence of clinically or practically meaningful differences.

## 4. Discussion

The aim of this study was to systematically assess ICU nurses’ knowledge, sustainability-related behaviors, and perceived barriers related to environmental sustainability using the preliminary version of the KABQES-ICU instrument, in order to provide empirical evidence on how sustainability is currently understood and enacted within intensive care practice. By assessing knowledge, behaviors, perceived barriers, and participant-reported organizational initiatives, this study sought to generate actionable insights to inform educational strategies, organizational policies, and behavior-change interventions, addressing a critical gap in the literature where sustainability initiatives often remain disconnected from frontline clinical realities [8,28].

A first consideration concerns the characteristics of the sample. Although the study included participants from several Italian regions, the distribution of participants was not homogeneous, with greater representation of nurses from central and northern regions of Italy. This imbalance may reflect differential engagement with professional networks and limits the extent to which the sample can be considered nationally representative. From a quantitative perspective, the associations of individual characteristics with sustainability-related outcomes appeared limited and heterogeneous across variables.

Educational attainment showed only limited associations with sustainability-related outcomes, and the observed effect sizes were small. This finding indicates that, within the participating sample, formal educational qualifications were not strongly differentiated according to the sustainability-related domains assessed. The previous literature has highlighted the still-variable integration of environmental sustainability into healthcare and nursing education [9,29], but the present study did not assess the sustainability content of participants’ educational programmes. Therefore, the observed differences cannot be attributed to educational exposure, and alternative explanations, including differences in age, professional experience, workplace context, or other unmeasured characteristics, cannot be excluded.

Professional role showed limited associations with sustainability-related outcomes. Although nurse coordinators reported lower perceived workload, time-pressure, organizational, and support barriers, the magnitude of this difference was small. Moreover, the present unadjusted analyses cannot determine whether this difference is related to professional responsibilities, organizational positioning, workplace characteristics, or other factors. No statistically significant differences were observed in sustainability knowledge or behaviors.

Age and professional experience showed several statistically significant but weak associations with sustainability-related scores. The magnitude of these correlations was modest, indicating limited shared variation between these characteristics and the sustainability domains. Although previous research has considered professional experience as potentially relevant to sustainability-related awareness and practice [10,18,25], the present cross-sectional and unadjusted analyses cannot establish whether experience contributes to these differences or whether the associations reflect age, workplace characteristics, previous exposure to sustainability-related information, or other unmeasured factors. These findings should therefore be considered descriptive and hypothesis-generating rather than explanatory.

Importantly, most statistically significant associations were small in magnitude. Correlations in the range of approximately 0.14–0.23 indicate limited shared variance between constructs, and the small effect sizes observed in subgroup comparisons suggest limited practical differences. Given the number of unadjusted secondary analyses performed, these findings should be interpreted primarily according to their magnitude and consistency rather than statistical significance alone.

Climate change knowledge scores were generally high, whereas reported sustainability behaviors were more heterogeneous. Knowledge was positively associated with both home- and work-based behaviors, but the correlations were weak (r = 0.22–0.23), indicating that only a limited proportion of variability in reported behaviors was shared with knowledge. This pattern is compatible with the broader literature suggesting that knowledge alone may not correspond closely to pro-environmental behavior [10,11]. However, the present findings do not demonstrate a mechanism linking knowledge to behavior and do not establish that the observed discrepancy was attributable to workload, time pressure, organizational support, or other contextual conditions. Although such factors have been identified as relevant in previous critical-care and healthcare sustainability research [13,15,24,25], their independent or mediating contribution was not tested in the present study. The observed pattern should therefore be regarded as hypothesis-generating and warrants examination using longitudinal and multivariable designs.

The organizational-initiative checklist provides descriptive information on participants’ awareness but cannot establish whether specific initiatives were objectively implemented within their organizations. Awareness varied substantially across initiatives, and nearly one third of participants reported being unaware of any sustainability-related initiatives. Because institutional affiliation was not collected and organizational practices were not independently verified, these findings cannot distinguish between limited implementation and limited staff awareness or communication. They should therefore be interpreted only as contextual descriptive information. This distinction is particularly important when considering international recommendations that emphasize coordinated organizational and system-level approaches to environmental sustainability in critical care [13,25,27].

These results are consistent with international evidence showing that healthcare professionals frequently report workload, time pressure, and limited organizational support as barriers to the implementation of sustainable practices [14,15]. While the patterns observed in this study appear broadly aligned with those reported in other healthcare systems, the uneven regional distribution of the sample and the web-based sampling approach suggest that contextual and territorial factors should be further explored.

Taken together, the findings describe a sample characterized by high climate change knowledge, moderate reported sustainability behaviors, moderate-to-high perceived barriers, and variable awareness of organizational sustainability initiatives. The associations among these dimensions were generally modest and were derived from unadjusted bivariate analyses. Accordingly, the coexistence of these characteristics should not be interpreted as evidence that perceived organizational barriers explain reported behaviors or that individual and organizational factors operate through specific pathways. The present data cannot determine temporal ordering, mediation, confounding, or the relative contribution of these factors. Rather, the observed patterns identify relationships that may be examined more directly in future longitudinal, multivariable, and multilevel studies.

Future research should therefore move beyond cross-sectional analyses and adopt longitudinal and multilevel designs to better understand the mechanisms linking knowledge, organizational constraints, and behavior. Particular attention should be given to geographical representation and organizational context, as well as to the development and evaluation of integrated interventions combining educational and system-level strategies to support the implementation of sustainable practices in intensive care. Future studies should also use the finalized KABQES-ICU structure and examine whether the findings can be replicated in independent samples in Italy and other countries.

### 4.1. Limitations and Strengths

While this study provides insights into sustainability-related practices in intensive care, several limitations should be acknowledged. First, the use of self-reported measures may have resulted in overestimation of both knowledge and behaviors due to social desirability bias. Second, all constructs were assessed through the same self-report survey, which may have introduced common method bias and inflated some of the observed associations [30]. Although anonymity and different response anchors were used as procedural safeguards, these measures do not eliminate common-source measurement effects, and no post hoc diagnostic can conclusively exclude this source of bias. Consequently, the magnitude of some observed correlations may partly reflect shared measurement method or response tendencies rather than substantive relationships between the underlying constructs, although the extent of this influence cannot be quantified from the present data. Third, the voluntary and open-access nature of participation may have introduced selection and volunteer bias, with greater participation from individuals already interested in sustainability. This self-selection may therefore have resulted in higher observed knowledge and sustainability-behavior scores than would be expected in the broader population of Italian ICU nurses, further limiting the generalizability of the estimates. Because recruitment occurred through the ANIARTI website and social media channels, the number and characteristics of individuals potentially reached, those who viewed the invitation, and those who accessed or started the survey without submitting it were not recorded. A conventional response rate could therefore not be calculated, respondents could not be compared with non-respondents, and potential non-response bias could not be directly assessed. Fourth, the uneven regional distribution of participants, with greater representation from central and northern Italy, may limit the national representativeness of the sample. The absence of an a priori sample-size or precision calculation limits the ability to determine whether the achieved sample provided adequate precision for all estimates or adequate statistical power for the secondary analyses, particularly subgroup comparisons involving sparsely represented categories. Fifth, the study was conducted within the Italian healthcare context, which may limit the transferability of the findings to healthcare systems with different organizational, cultural, and policy contexts. Sixth, organizational sustainability initiatives were assessed through a participant-reported multiple-response checklist and were not independently verified at the institutional level. Non-selection of an initiative could reflect either its absence or limited participant awareness and therefore cannot be interpreted as evidence that the initiative was not implemented. Accordingly, these findings should be interpreted as participants’ reported awareness of organizational initiatives rather than as objective estimates of their presence, absence, or implementation within healthcare organizations. In addition, organizational affiliation was not collected, and multiple participants may have referred to the same healthcare organization; institutional clustering could therefore not be identified or accounted for. Seventh, the statistical analyses were primarily descriptive and bivariate. They were appropriate for the exploratory baseline objective of the study but do not allow the identification of independent associations or the exclusion of residual confounding. Because multiple unadjusted secondary comparisons were performed, the possibility of type I error cannot be excluded. In addition, the cross-sectional design does not establish temporal ordering among knowledge, reported behaviors, and perceived barriers. Reverse directionality and residual or unmeasured confounding therefore cannot be excluded. For example, greater engagement in sustainable practices may increase participants’ knowledge or awareness, rather than knowledge necessarily preceding behavior.

Finally, the survey was conducted and originally analyzed using the preliminary 33-item, four-domain version of the KABQES-ICU. A subsequent psychometric study, published after the initial submission of this manuscript, refined the questionnaire into a 28-item, five-factor instrument [19]. Five items were removed, and the preliminary combined barrier domain was subsequently differentiated into workload and time-pressure barriers and organizational and support barriers. These modifications may have affected the content coverage, score distributions, and magnitude of associations observed in the present study. In particular, combining two subsequently differentiated barrier dimensions into a single score may have obscured distinct patterns of association with knowledge and behaviors. Therefore, the domain scores and correlations reported in this baseline study should not be considered directly equivalent to those that would be obtained using the finalized KABQES-ICU, and replication using the validated structure is warranted.

Despite these limitations, this study has several important strengths. It provides a structured and multidimensional baseline assessment of environmental sustainability-related knowledge, behaviors, and perceived organizational barriers among ICU nurses. The use of a context-specific instrument allowed for a more context-specific assessment of the clinical and organizational dynamics of intensive care settings. The inclusion of participants from multiple Italian regions provides information from different areas of the country, although the self-selected and unevenly distributed sample does not provide nationally representative estimates. By describing the relationships among knowledge, behaviors, barriers, and professional characteristics, this study extends previous qualitative work in this field and provides a foundation for future longitudinal and intervention-based research.

### 4.2. Implications for Clinical Practice

The present descriptive findings do not establish which educational or organizational interventions would be most effective. However, they identify areas that warrant evaluation in future intervention studies, including professional education, workload and time constraints, organizational support, and the communication of existing sustainability initiatives. Although climate change knowledge was positively associated with reported sustainability behaviors, these associations were modest and unadjusted and should not be interpreted as evidence of an independent effect.

The observed levels of workplace behaviors and perceived barriers identify organizational conditions as an area requiring further investigation. However, the present analyses do not establish whether workload, time pressure, staffing, resource availability, or organizational support independently affect sustainability-related behaviors. Future intervention studies should directly test whether modifying these conditions improves sustainable clinical practice.

Accordingly, the present findings do not establish which interventions should be implemented, but they identify organizational barriers and the communication of sustainability initiatives as potentially relevant targets for future evaluation. The proportion of participants who selected “I am not aware of any sustainability-related initiatives” warrants further investigation but does not establish whether such initiatives were absent or insufficiently communicated.

## 5. Conclusions

This study provides exploratory baseline evidence on environmental sustainability-related knowledge, behaviors, and perceived barriers among participating Italian ICU nurses. High climate change knowledge scores coexisted with moderate reported sustainability behaviors, substantial perceived barriers, and limited reported awareness of several organizational sustainability initiatives. Associations between knowledge and reported behaviors were modest and unadjusted. The findings therefore do not establish causality, temporal direction, independent predictors, or the relative contribution of individual and organizational factors. Future longitudinal and multilevel studies using the finalized KABQES-ICU and independently verified organizational data are needed to investigate these relationships.

## Figures and Tables

**Table 1:** Sociodemographic and professional characteristics of the study sample.

Characteristic	Category/Statistic	Value
**Sociodemographic characteristics**		
Age	Mean ± SD	40.47 (10.32)
Gender identity	Male	93 (33.5)
	Female	180 (64.7)
	Non-binary	1 (0.4)
	Not declared	4 (1.4)
**Professional characteristics**		
Clinical experience, years	Mean ± SD	16.05 (10.15)
ICU experience, years	Mean ± SD	10.98 (9.23)
Professional role	Clinical nurse	243 (87.4)
	Nurse coordinator	32 (11.59)
	Not declared	3 (1.1)
**Geographic distribution**		
Abruzzo		2 (0.7)
Calabria		5 (1.8)
Campania		13 (4.7)
Emilia-Romagna		29 (10.4)
Friuli-Venezia Giulia		1 (0.4)
Lazio		57 (20.5)
Liguria		7 (2.5)
Lombardia		31 (11.2)
Piemonte		30 (10.8)
Puglia		15 (5.4)
Sardegna		13 (4.7)
Sicilia		5 (1.8)
Toscana		34 (12.2)
Trentino-Alto Adige		2 (0.7)
Umbria		7 (2.5)
Valle d’Aosta		1 (0.4)
Veneto		24 (8.6)
Not declared		2 (0.7)

Note. Values are reported as n (%) unless otherwise specified. Percentages were calculated on the total sample unless otherwise indicated. ICU = intensive care unit; SD = standard deviation.

**Table 2:** Organizational sustainability initiatives selected by participants in their workplace organizations.

Which Sustainability Initiatives Are Implemented by the Organization Where You Work?	n (%)
Annual reporting of carbon emissions	5 (1.8)
Development of plans and strategies to reduce carbon emissions	13 (4.7)
Construction of new buildings or spaces based on sustainability criteria	38 (13.7)
Renovation or upgrading of existing buildings or spaces according to sustainability criteria	46 (16.5)
Smart monitoring and control of air conditioning and heating systems	57 (20.5)
Smart monitoring and control of water use	39 (14.0)
On-site renewable energy and heat production (e.g., removal of coal and oil heating systems and use of photovoltaic panels)	21 (7.6)
Use of electric, hybrid, or low-carbon vehicles	44 (15.8)
Promotion of healthier and more active transportation (e.g., cycling and walking)	45 (16.2)
Reduction in travel through digital redesign of care pathways (telemedicine)	29 (10.4)
Reduction in single-use plastic consumption	57 (20.5)
Reduction in paper use	47 (16.9)
Reduction in the use of single-use materials and devices	33 (11.9)
Sterilization and reuse of metal instruments and other devices	134 (48.2)
Reduction in food waste	49 (17.6)
Transition to plant-based diets (use of seasonal and local products)	14 (5.0)
Digitization of health records	129 (46.4)
Selection of suppliers that are decarbonizing their processes	5 (1.8)
Staff training on sustainability best practices	33 (11.9)
I am not aware of any	85 (30.6)

Note. Multiple responses were allowed; therefore, percentages do not sum to 100%. Values represent the number and percentage of participants who selected each initiative. Non-selection should not be interpreted as evidence that an initiative was absent, because the checklist assessed participants’ reported awareness. The organizational initiative checklist was developed by the research team on the basis of international sustainable healthcare recommendations and the ICU-specific sustainability literature [25,27]. It was used descriptively and was not treated as a validated measurement scale.

**Table 3:** Descriptive statistics and distribution characteristics of domain scores.

Domain Score	N	Mean ± SD	Skewness (SE)	Kurtosis (SE)
Climate Change Knowledge	278	79.68 ± 16.21	−1.06 (0.15)	1.43 (0.29)
Home-Based Sustainability Behaviors	278	63.20 ± 15.56	−0.14 (0.15)	0.05 (0.29)
Work-Based Sustainability Behaviors	278	59.63 ± 15.59	0.09 (0.15)	0.87 (0.29)
Workload, Time Pressure, Organizational & Support Barriers	278	63.18 ± 18.65	−0.24 (0.15)	0.04 (0.29)

Note. SD = standard deviation; SE = standard error.

**Table 4:** Pearson correlations among standardized domain scores.

		Climate Change Knowledge	Home-Based Sustainability Behaviors	Work-Based Sustainability Behaviors	Workload, Time Pressure, Organizational & Support Barriers
Climate Change Knowledge	Pearson Correlation	1	0.22 **	0.23 **	0.15 *
Home-Based Sustainability Behaviors	Pearson Correlation	0.22 **	1	0.55 **	0.14 *
Work-Based Sustainability Behaviors	Pearson Correlation	0.23 **	0.55 **	1	0.14 *
Workload, Time Pressure, Organizational & Support Barriers	Pearson Correlation	0.15 *	0.14 *	0.14 *	1

Note. Values are Pearson correlation coefficients (r). N = 278 for all correlations. All analyses were conducted using two-tailed tests. * *p* < 0.05; ** *p* < 0.01.

## Data Availability

The data that support the findings of this study are not publicly available due to privacy and ethical restrictions, as participants provided informed consent for the use of anonymized data for the purposes of the present research. Anonymized aggregated data may be made available from the corresponding author upon reasonable request and subject to approval by the research team and applicable ethical and data protection regulations.

## Abbreviations

The following abbreviations are used in this manuscript:

ANIARTI	Italian National Association of Critical Care Nurses
ANOVA	Analysis of Variance
CCK	Climate Change Knowledge
ICU	Intensive Care Unit
SB_Home	Home-Based Sustainability Behaviors
SB_Work	Work-Based Sustainability Behaviors
KABQES-ICU	Knowledge, Attitudes, and Behaviors Questionnaire on Environmental Sustainability in Intensive Care Units
SD	Standard Deviation
SE	Standard Error
SPSS	Statistical Package for the Social Sciences
STROBE	Strengthening the Reporting of Observational Studies in Epidemiology
WHO	World Health Organization
WTPOSB	Workload, Time Pressure, Organizational & Support Barriers

## Appendix A

**Table A1:** Measurement items, response options, scoring, and sources of the preliminary 33-item KABQES-ICU.

Domain	Item No.	Exact Item Administered	Response Options	Scoring	Item Provenance/Source
Climate Change Knowledge	CC_1	The planet has warmed significantly since 1850, leading to climate change.	0 = Not at all familiar; 1 = Slightly familiar; 2 = Moderately familiar; 3 = Very familiar; 4 = Extremely familiar	Raw sum of 5 items (0–20); subsequently rescaled to 0–100. Higher scores indicate greater climate change knowledge.	Author-generated; informed by previous qualitative findings [8], the M-COM-B framework [20], and the ICU sustainability literature [13,15,24,25].
	CC_2	Climate change is largely caused by human behaviors that add greenhouse gases to the atmosphere.			
	CC_3	The healthcare system is responsible for approximately 8.5% of greenhouse gas emissions in the United States that contribute to global warming.			
	CC_4	Climate change increases the likelihood of adverse health conditions.			
	CC_5	Vulnerable populations and other at-risk groups experience greater negative health impacts due to climate change.			
Home-Based Sustainability Behaviors	IS_H_1	Use non-fossil-fuel energy sources (e.g., wind or solar energy, geothermal, purchase of offset energy, etc.).	0 = Never; 1 = Rarely; 2 = Sometimes; 3 = Often; 4 = Always	Raw sum of 5 items (0–20); subsequently rescaled to 0–100. Higher scores indicate more frequent home-based sustainability behaviors.	Author-generated; informed by previous qualitative findings [8], the M-COM-B framework [20], and evidence concerning pro-environmental behavior across home and work settings [21,22].
	IS_H_2	Save energy (e.g., using energy-efficient devices, maintaining moderate temperature settings, turning off lights and electronic devices, etc.).			
	IS_H_3	Use less gasoline (e.g., driving fuel-efficient vehicles, avoiding unnecessary trips, walking/biking, etc.).			
	IS_H_4	Reduce waste (e.g., buying less, reusing more, recycling, etc.).			
	IS_H_5	Choose foods that require fewer resources to grow/produce (local, seasonal, fewer animal products, less packaging).			
Work-Based Sustainability Behaviors	IS_W_1	Waste segregation (plastic, paper, glass, etc.).	0 = Never; 1 = Rarely; 2 = Sometimes; 3 = Often; 4 = Always	Raw sum of 8 items (0–32); subsequently rescaled to 0–100. Higher scores indicate more frequent work-based sustainability behaviors.	Author-generated; informed by previous qualitative findings [8] and the literature on sustainable ICU and nursing practice [13,15,24,25].
	IS_W_2	Energy saving (e.g., turning off lights and electronic devices).			
	IS_W_3	Reduction in noise pollution (e.g., intelligent alarm management).			
	IS_W_4	Commuting to work using active, shared, or public transportation (e.g., walking, biking, car-sharing, public transport).			
	IS_W_5	Waste reduction (using materials, devices, and medications appropriately proportioned).			
	IS_W_6	Recycling and reuse of materials and devices after reprocessing.			
	IS_W_7	Promoting the use of sustainable materials or devices over others.			
	IS_W_8	Asking workplace leadership to support policies, products, and/or processes that generate fewer greenhouse gas (GHG) emissions.			
Workload, Time Pressure, Organizational and Support Barriers	C_1	Poor logistics (lack of waste-segregation containers or inconvenient placement).	0 = Not at all; 1 = Slightly; 2 = Moderately; 3 = Very; 4 = Extremely	Raw sum of 15 items (0–60); subsequently rescaled to 0–100. Higher scores indicate greater perceived barriers.	Author-generated; informed by previous qualitative findings [8], M-COM-B opportunity constructs [20], and the literature on workload and organizational barriers in sustainable critical care [13,15,24,25].
	C_2	Physical and mental fatigue.			
	C_3	Insufficient human resources.			
	C_4	Limited availability of materials and devices.			
	C_5	Materials and devices not appropriate or proportionate to individual cases.			
	C_6	Excessive workload.			
	C_7	Lack of time during routine work shifts.			
	C_8	Lack of time during emergency or urgent situations.			
	C_9	Lack of knowledge and training.			
	C_10	Conflict between patient safety and sustainability.			
	C_11	Infection risk.			
	C_12	Lack of organizational support.			
	C_13	Lack of peer support.			
	C_14	Absence of protocols, procedures, guidelines, or regulations.			
	C_15	Higher costs.			

Note. The preliminary psychometric section administered in the present study comprised 33 items: 5 climate change knowledge items, 5 home-based sustainability behavior items, 8 work-based sustainability behavior items, and 15 perceived-barrier items. The separate organizational sustainability initiative checklist was descriptive and was not part of the 33-item psychometric scoring structure. The preliminary 33-item version should be distinguished from the subsequently validated 28-item, five-factor KABQES-ICU [19].
